# Antibacterial and Antitumor Potential of Actinomycetes Isolated from Mangrove Soil in the Maowei Sea of the Southern Coast of China

**Published:** 2018

**Authors:** Bin Gong, Shuang Chen, Wenwen Lan, Yanmin Huang, Xiangcheng Zhu

**Affiliations:** a *Guangxi Key* *Laboratory of Beibu Gulf Marine Biodiversity Conservation, Ocean Science department, Qinzhou University, Qinzhou, China. *; b *College of Chemistry and Materials Science, Guangxi Teachers Education University, Nanning, China.*; c *Xiangya International Academy of Translational Medicine, Central South University, Changsha, China.*

**Keywords:** Antibacterial, Antitumor, Actinomycetes, Mangrove, China

## Abstract

Mangroves are the tidal forest existing in the intertidal zone and usually considered as the special marine ecosystem. In the present study, 452 actinomycetes were recovered from nine diferent sites at Maowei Sea Mangrove Reserve in Qinzhou (Guangxi province, China). Among them, Seventy-four strains were purified for 16s RNA gene sequencing and further characterization. The results indicated that the majority of isolates belonged to the genera *Streptomyces, *including 17 species. *Streptomyces sanyensis* was the dominant species (31.1%), followed by *Streptomyces griseorubens* (17.5%), *Streptomyces viridobrunneus* (10.8%) and other *Streptomyces *species. Only one rare actinomycete, *Stenotrophomonas *was discovered*. *The isolation of actinomycetes was obviously related to the type of soil and edaphic conditions. Rhizosphere-associated soils gave almost 62.2% actinomycete isolates, nearly twice as many as the non-rhizosphere-associated soils. In addition, 20 actinomycete strains (27%) presented varied antibacterial activities towards four tested organisms, including two drug-resistant clinical strains (MRSA and VRE), while some species of *Streptomyces* like *S.sanyensis*, *S.viridobrunneus*, *S.tanashiensis*, *S.parvus*, *S.flavotricini,* and *S.parvulus* exhibited remarkable cytotoxic activities. Further bioinformatical analysis of these 29 bioactive strains for secondary metabolites biosynthetic machineries revealed that nonribosomal peptide synthetase (NRPS) was detected in 20 isolates (68.9%), whereas type-I polyketide synthase (PKS-I) and type-II polyketide synthase (PKS-II) were detected in 16 and all of the 29 strains, respectively. Hence, our work demonstrated that actinomycetes from mangroves in Maowei Sea Mangrove Reservewere fascinating reservoirs for antibacterial and antitumor natural products discovery.

## Introduction

Cancer and multiple drug-resistant (MDR) bacteria continue to be emerging global-scale intractable problems (1, 2), therefore developing new drugs to combat those threats is an urgent need for today′s pharmaceutical compendium. Actinomycetes have been studied for more than 60 years, yielding many important anti-cancer, anti-infection, and other bioactive natural products. Among roughly 33, 500 known bioactive metabolites from bacteria, 40% were produced by actinobacteria, and more than 10, 400 were reported from the actinomycetes genus *Streptomyces* (3). However, the discovery of novel natural products from terrestrial actinomycetes was stagnant after the long term exploration, and marine actinomycetes have attracted considerable attention recently (4, 5).

Mangroves are tidal forest existing in the intertidal zone and usually considered as special marine ecosystem. Because of its unique environmental conditions such as high moisture and high salinity, organisms from mangrove differ greatly from terrestrial and thus could produce novel metabolites. In the recent years, studies on mangrove derived actinomycetes (mainly isolated from sediments and mangrove plants) and their secondary metabolites have become a hot spot. The occurrences of actinomycetes from mangrove habitats have been reported in China (6, 7), India (8), Iran (9) Thailand (10) Malaysia (11) and so on. Up to date the 24 genera of 11 families and eight suborders under the actinomycetales containing three new genera have been isolated and identified from mangrove (12).

In China, mangroves commonly grow on the coast of southeast China, in the provinces of Hainan, Guangdong, Guangxi, Fujian and Taiwan. Among them, Guangxi possesses the largest mangrove forest in China, with the total area of about 8374.9 hm^2^, and 15 mangrove plants species documented (13). So far, two National Mangrove Reserves have been established in Guangxi province: Shankou and Beilun. Because of the comparatively rare exploitation, mangrove actinomycetes in Guangxi province could be a potential source for the discovery of new species and novel natural products (14, 15). 

The primary goal of this research was to investigate the diversity and distribution of culturable actinomycetes from relatively unexplored regions of Maowei Sea Mangrove Reserve and evaluate their antibacterial and cytotoxic activities. The presence of biosynthetic machineries including PKS-I, PKS-II and NRPS in our screened bioactive strains suggested the great potential of mangrove-derived actinomycetes in the discovery of novel natural products. 

## Experimental

*Sample collection and strain isolation*

The sediments were collected from different sites of Maowei Sea Mangrove Reserve (Qinzhou, Guangxi province, China) in May 2013 (Table 1). Each sample was ascetically transferred to a dry and sterile plastic bag, then air-dried and grounded with a mortar and pestle. One gram of such soil sample was mixed with 5 mL sterilized water, and spread onto the solid Gause I medium (soluble starch 20 g, KNO_3_ 1 g, NaCl 0.5 g, K_2_HPO_4_ 0.5 g, MgSO_4_ 0.5 g, FeSO_4_ 0.01 g, artificial sea water (ASW) 1000 mL, agar 15 g) supplemented with cycloheximide (100 μg mL^-1^) and nystatin (25 μg mL^-1^), and incubated at 27 ± 2 ℃ for 14 days. The resulting actinomycete isolates were further purified using M1 medium (16), which contains 10 g of starch, 4 g of yeast extract, 2 g of peptone,15 g of agar, and 1000 mL of ASW.

**Table 1:** The characteristic of soils in sampling sites

**Sampling sites**	**The characteristic of soil**	**Sampling depth**
Site 1	Rhizosphere of *Excoecaria agallocha*	1~5 cm under soil surface
Site 2		10~15 cm under soil surface
Site 3	Rhizosphere of *Kandelia candel*	1~5 cm under soil surface
Site 4		10~15 cm under soil surface
Site 5	sediment	10~15 cm under soil surface
Site 6	sediment	10~15 cm under soil surface
Site 7	sediment	10~15 cm under soil surface
Site 8	Sandy soil	10~15 cm under soil surface
Site 9	Small stones soil mixed with clay	10~15 cm under soil surface

*DNA extraction and 16S rRNA analyses of selected strains*

For each isolated target strain, its genomic DNA was extracted following the method of Magarvey (17) and 16S rRNA gene was amplified using the universal primers: 27f (5’-AGAGTTGATCCPATGGCTCAG-3’) and 1541R (5’-AAGGAGGTGATCCAGCC-3’) (18). The PCR program was carried out under following conditions: initial denaturation at 95℃ for 4 min; 35 cycles of 94℃ for 1 min, 52℃ for 1 min, and 72℃ for 1.5 min; and a final extension at 72℃ for 8 min. The PCR product was purified using QIAquick gel extraction kit (Qiagen, Hilden, Germany), then sequenced by the GenScript Company Ltd (Nanjing, China). The obtained 16S rRNA sequence was searching for the closest match sequences from GenBank by BLASTn. The resulting sequences were aligned using the ClustalX, and the MEGA 5.0 was employed to construct the phylogenetic tree by using the Maximum Likelihood (ML) method under the Kimura two parameter model and bootstrap analyses with 1,000.

*Detection of secondary metabolites biosynthetic machineries*

To evaluate the biosynthetic potential of isolated bioactive actinomycete strains, the genes encoding nonribosomal peptide synthetase (NRPS), type-I polyketide synthase (PKS-I) and type-II polyketide synthase (PKS-II) were amplified by using three sets of primers including A3F (5’-GCSTACSYSATSTACACSTCSGG-3’) and A7R (5’-SASGTCVCCSGTSCGGTAS-3’), K1F (5’-TSAAGTCSAACATCGGBCA-3’) and M6R (5’-CGCAGGTTSCSGTACCAGTA-3’), KsaF (5’-TSGCSTGCTTGGAYGCSATC-3’) and KsaR (5’-TGGAANCCGCCGAABCCGCT-3’), respectively (19). Negative control without DNA template was properly set. PCR products purification, sequencing and sequence analyses were performed as described previously. 

*Bioactive assays of isolated strains*

For antibacterial assay, two common strains *Bacillus cereus* ATCC 14579 and *Escherichia coli *ATCC 13706 (they were purchased from China Center of Industrial Culture Collection and preserved in our lab) and two drug-resistant clinical strains *Staphylococcus aureus *SA115 and *Enterococcus faecium* EF009 (They were kindly offered by clinical laboratory of the First People's Hospital in Qinzhou) were applied for screening tests as described earlier (20). After 7 to 12 days cultivation, the fermentation broth of target strains were centrifuged and supernates were used for antibacterial activity screening using the paper disk (diameter, 6 mm) assay method. The inhibition zone of broth sample was measured and compared with the positive and negative controls to evaluate the antibacterial activity of isolated strains. Each test was carried out intriplicate. 

 The cytotoxic activity of fermentation broth sample (diluted up to 200 times) was studied by the MTT assay using cell lines CNE-2 and Hela. The MTT assay protocol was adapted and the growth inhibition rate was calculated according to the method described by Mosmann (21). The fermentation broth of target strain was regarding cytotoxic when the growth inhibition rate of tested cell line was more than 60%.

## Results and Discussion

*Distribution of actinomycetes*

**Figure 1:**
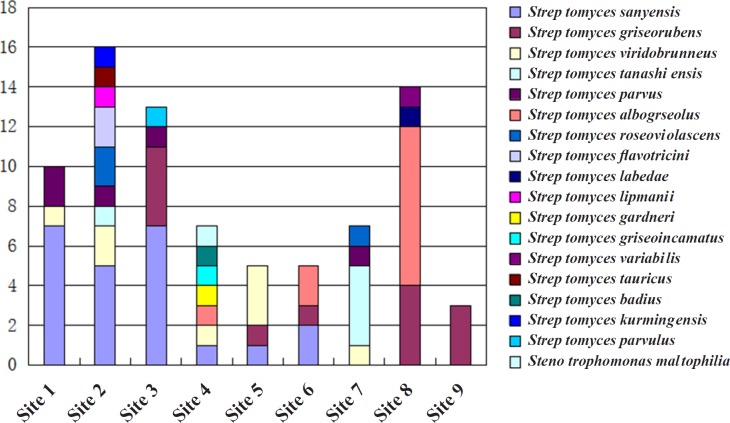
different species of actinomycetes in different mangrove soil

Based on the colonial morphology of viable isolates, 452 actinomycetes were recovered from the nine different sampling sites at Maowei Sea Mangrove Reserve, China. The preliminary morphological exhibition suggested that the most abundant genus of isolated actinomycetes was *Streptomyces*. 74 strais representing all purified clonies were subjected to 16s rRNA analyses, and only two genera *Streptomyces* and *Stenotrophomonas* were identified. *Streptomyces sanyensis* was the dominant species (23 clonies, 31.1%) followed by *Streptomyces griseorubens *(13 clonies, 17.5%), *Streptomyces viridobrunneus *(8 clonies, 10.8%) *Streptomyces tanashiensis *(5 clonies, 6.7%) *Streptomyces parvus *(5 clonies, 6.7%) *Streptomyces albogriseolus* (5 clonies, 6.7%) *Streptomyces roseoviolascens *(3 clonies, 4.0%) *Streptomyces flavotricini* (2 clonies, 2.7%) nine other *Streptomyces *species (one clony of each, total of 12.2%) and one *Stenotrophomonas maltophilia* species (Figure 1). The majority of analyzed strains were all belong to *Streptomyces.*

Distinct variations of actinomycetes counts ranged from 0.5 to 9.8% in the total bacterial population were observed among investigated habitats. The number of actinomycetes was obviously related to the type of soil and edaphic conditions. Rhizosphere-associated soils (sampling sites 1–4) gave almost 62.2% actinomycete isolates, nearly twice as many as the non-rhizosphere-associated soils (sampling sites 5–9). The type of species isolated from rhizosphere-associated soils (7–9 different species) were also conspicuously more than those from surface (usually 3–4 kinds of species). Although the diversity was relatively low, actinomycetes isolated from sandy soil were more than non-rhizosphere-associated soils and small stones soil mixed with clay (Figure 1). Our findings were consistent with the reports from other researchers. For example, Hong had isolated actinomycetes from soil and plant material of eight mangrove sites in China, and the highest average number of actinomycetes was obtained from the rhizosphere soil samples of Fujian mangrove (6).The distribution of actinomycetes in the Bay of Bengal (India) also indicated that the highest number of actinomycetes was isolated from an intertidal region having alluvial soil, and soil nitrogen was the key factor determining the antagonistic activity (22).

*The Antibacterial and cytotoxicity activities of isolated actinomycetes*

All selected 74 actinomycetes were assayed for their antagonistic behaviors against 4 test bacterial strains (two common strains *Bacillus cereus* ATCC 14579 and *Escherichia coli *ATCC 13706, two drug-resistant clinical strains *Staphylococcus aureus* SA115 and *Enterococcus Faecium* EF009). It was observed that 20 strains (27%) suppressed the growth of test organismsin in different degrees. As shown in Table 1, 18 strains (90%) were against Gram-positive bacteria and 9 strains (45%) were against Gram-negative bacteria, while 9 strains (45%) exhibited antimicrobial activity on both Gram-positive and Gram-negative bacteria. Three strains (one of *Streptomyces griseorubens* and two of *Streptomyces parvus*) showed good activity against drug-resistant clinical strains. Interestingly, the screened bioactive actinomycetes were all belonged to *Streptomyces,* which then could be classified into ten subgroups including *sanyensis* (10%) *griseorubens *(30%) *albogriseolus* (20%), *parvus* (10%) *flavotricini* (10%) *tanashiensis*, *labedae*, *parvulus*, *griseoincarnatus *and *variabilis*. And even strains from the same species (*S.sanyensis*, *S.griseorubens* and *S.albogriseolus*) presented dissimilar antibacterial spectrum (Table 1).

The cytotoxicity assay results showed that 12 strains (16.2%) displayed cytotoxic activity on CNE-2 cell line and 5 strains (6.7%) on Hela cell line, whereas *S.sanyensis*, *S.flavotricini*, *S.parvulus* and *Stenotrophomonas maltophilia* presented cytotoxic activity against both CNE-2 and Hela cell lines. Except one strain of *Stenotrophomonas*, others were primary belonging to *Streptomyces *(Table 1). The species of *Streptomyces* such as *S.sanyensis*, *S.viridobrunneus*, *S.tanashiensis*, *S.parvus*, *S.flavotricini* and *S.parvulus *exhibited a marked cytotoxic activity. Varied cytotoxic activities from the same species were also observed. 

The bioactivity assay results of our isolated strains were well agreed with the reported data. Such as *S.sanyensis* isolated from mangrove sediment of Sanya (China) exhibited cytotoxic activity against the human colon tumour cell line HCT-116 (23) and unlike the terrestrial derived *S.griseorubens* that possessed antifungal and antibacterial activities (24) the *S.griseorubens*strain isolated from the sea also revealed strong antitumor activity against Hela, KB and SMMC7721 cells (25). In our work, 5 bioactive strains related to *S.sanyensis* were recovered from four different sampling sites, with three possessing antitumor activity and two presenting anticontagious potential; while 5 isolated *S.griseorubens *strains exhibited strong anticontagious potential but no antitumor activity was observed. Our research also revealed the clear relationship between strain species and their bioactivity, because 22 of 29 bioactive strains were mostly isolated from the predominent species (Isolation rate > 6.7%). This was probably due to the beneficial transfer of secondary metabolites biosynthetic gene clusters among large population of certain species. Moreover, though the biodiversity of actinomycetes isolated from sites 6, 7 and 8, whose soils were more infertile, were less than those from other sampling sites, many strains derived from these infertilesites exhibited strong antitumor and antibacterial activities. Hence these mangrove soilsmay be relatively more preferred for isolation of bioactive actinomycetes.

*Detection of secondary metabolites biosynthetic machineries in selected actinomycetes*

The 29 bioactive strains were further screened for the possession of typical secondary metabolites biosynthetic machineries including PKS-I, PKS-II, and NRPS (Table 1). As the result, NRPS was detected in 20 isolates (68.9%) whereas PKS-I and PKS-II were detected in 16 and all of the 29 strains, respectively. More significantly, 11 strains (LS1-6, LS2-19, LS2-20, LS4-13, LS7-6, LS7-8, LS1-2, LS2-17, LS3-3, LS2-3 and LS2-14) had both PKS-II and NRPS, 7 strains (LS3-16, LS8-5, LS8-10, LS3-2, LS6-2, LS8-14 and LS8-3) had both PKS-I and PKS-II, and 9 strains (LS6-3, LS8-2, LS8-9, LS8-13, LS2-21, LS6-1, LS3-8, LS4-7 and LS8-4) possessed all three target biosynthetic machineries. These findings logically explained the great potential of our isolated bioactive strains. 

**Table 2:** Cytotoxic activity and antimicrobial activity profile of the Actinobacteria isolates

**Isolate**	**Putative species**	**Antimicrobial activity**	**Cytotoxic activity**	Presence of gene						
		***Bacillus cereus*** ***ATCC 14579***	***Escherichia coli*** ***ATCC 13706***	***Staphylococcus aureus*** ** SA115** a*	***Enterococcus Faecium*** ** EF009** ^ a*^	**CNE-2**	**Hela**	PKS-I	PKS-II	NRPS
LS1-6	*Streptomyces sanyensis*	-	-	-	-	+	-	-	+	+
LS2-19	*Streptomyces sanyensis*	-	-	-	-	+	-	-	+	+
LS2-20	*Streptomyces sanyensis*	-	-	-	-	+	+	-	+	+
LS5-4	*Streptomyces sanyensis*	+	-	-	-	-	-	-	+	-
LS3-16	*Streptomyces sanyensis*	+++	+	-	-	-	-	+	+	-
LS6-3	*Streptomyces griseorubens*	+	-	-	-	-	-	+	+	+
LS8-2	*Streptomyces griseorubens*	+++	+	-	-	-	-	+	+	+
LS8-5	*Streptomyces griseorubens*	+++	+	-	-	-	-	+	+	-
LS8-9	*Streptomyces griseorubens*	+++	+	-	-	-	-	+	+	+
LS8-10	*Streptomyces griseorubens*	+++	+	-	-	-	-	+	+	-
LS8-13	*Streptomyces griseorubens*	-	-	-	+	-	-	+	+	+
LS2-21	*Streptomyces viridobrunneus*	-	-	-	-	+	-	+	+	+
LS4-13	*Streptomyces viridobrunneus*	-	-	-	-	+	-	-	+	+
LS7-6	*Streptomyces tanashiensis*	+++	-	-	-	-	-	-	+	+
LS7-8	*Streptomyces tanashiensis*	-	-	-	-	+	-	-	+	+
LS1-2	*Streptomyces parvus*	-	-	+	-	-	-	-	+	+
LS2-17	*Streptomyces parvus*	-	-	+	-	+	-	-	+	+
LS3-3	*Streptomyces parvus*	-	-	-	-	+	-	-	+	+
LS3-2	*Streptomyces albogriseolus*	+	+	-	-	-	-	+	+	-
LS6-2	*Streptomyces albogriseolus*	+	-	-	-	-	-	+	+	-
LS6-1	*Streptomyces albogriseolus*	+	-	-	-	-	-	+	+	+
LS8-14	*Streptomyces albogriseolus*	+	+	-	-	-	-	+	+	-
LS2-3	*Streptomyces flavotricini*	+	+	-	-	+	+	-	+	+
LS2-14	*Streptomyces flavotricini*	+	+	-	-	+	+	-	+	+
LS8-3	*Streptomyces labedae*	+	-	-	-	-	-	+	+	-
LS3-8	*Streptomyces parvulus*	+++	-	-	-	+	+	+	+	+
LS4-2	*Stenotrophomonas maltophilia*	-	-	-	-	+	+	-	+	-
LS4-7	*Streptomyces griseoincarnatus*	+	-	-	-	-	-	+	+	+
LS8-4	*Streptomyces variabilis*	+	-	-	-	-	-	+	+	+

a* **: **multiple drug resistance strains;

## Conclusion

In our research, the regular distribution of actinomycetes was different aried mangrove soil sites, and the largest diversity of actinomycetes was isolated from rhizosphere-associated soils. Among all isolated strains from mangrove soil, the most predominant species were *Streptomyces sanyensis *and *Streptomyces griseorubens. *The subsequent bioassays have screened out 29 bioactive strains possessing varied antibacterial and antitumor potentials, which were recovered from different soil samples. Further bioinformatical analyses have revealed the enrichment of secondary metabolites biosynthetic machineries in these bioactive strains. Though many isolated bioactive strains mentioned above have been reported before, some of them were rarely derived from marine envionment. Thus, the present study demonstrated that actinomycetes from mangrove in Maowei Sea Mangrove Reserve were abundant resources for novel antibacterial and antitumor natural products discovery. 
